# Entity recognition from clinical texts via recurrent neural network

**DOI:** 10.1186/s12911-017-0468-7

**Published:** 2017-07-05

**Authors:** Zengjian Liu, Ming Yang, Xiaolong Wang, Qingcai Chen, Buzhou Tang, Zhe Wang, Hua Xu

**Affiliations:** 1 Key Laboratory of Network Oriented Intelligent Computation, Harbin Institute of Technology Shenzhen Graduate School, Shenzhen, 518055 China; 2 Pharmacy Department, Shenzhen Second People’s Hospital, First Affiliated Hospital of Shenzhen University, Shenzhen, 518035 China; 3 Key Laboratory of Symbolic Computation and Knowledge Engineering of Ministry of Education, Jilin University, Changchun, 130012 China; 4 School of Biomedical Informatics, The University of Texas Health Science Center at Houston, Houston, TX USA

**Keywords:** Entity recognition, Recurrent neural network, Clinical notes, Deep learning, Sequence labeling

## Abstract

**Background:**

Entity recognition is one of the most primary steps for text analysis and has long attracted considerable attention from researchers. In the clinical domain, various types of entities, such as clinical entities and protected health information (PHI), widely exist in clinical texts. Recognizing these entities has become a hot topic in clinical natural language processing (NLP), and a large number of traditional machine learning methods, such as support vector machine and conditional random field, have been deployed to recognize entities from clinical texts in the past few years. In recent years, recurrent neural network (RNN), one of deep learning methods that has shown great potential on many problems including named entity recognition, also has been gradually used for entity recognition from clinical texts.

**Methods:**

In this paper, we comprehensively investigate the performance of LSTM (long-short term memory), a representative variant of RNN, on clinical entity recognition and protected health information recognition. The LSTM model consists of three layers: input layer – generates representation of each word of a sentence; LSTM layer – outputs another word representation sequence that captures the context information of each word in this sentence; Inference layer – makes tagging decisions according to the output of LSTM layer, that is, outputting a label sequence.

**Results:**

Experiments conducted on corpora of the 2010, 2012 and 2014 i2b2 NLP challenges show that LSTM achieves highest micro-average F1-scores of 85.81% on the 2010 i2b2 medical concept extraction, 92.29% on the 2012 i2b2 clinical event detection, and 94.37% on the 2014 i2b2 de-identification, which is considerably competitive with other state-of-the-art systems.

**Conclusions:**

LSTM that requires no hand-crafted feature has great potential on entity recognition from clinical texts. It outperforms traditional machine learning methods that suffer from fussy feature engineering. A possible future direction is how to integrate knowledge bases widely existing in the clinical domain into LSTM, which is a case of our future work. Moreover, how to use LSTM to recognize entities in specific formats is also another possible future direction.

## Background

With rapid development of electronic medical record (EMR) systems, more and more EMRs are available for researches and applications. Entity recognition, one of the most primary clinical natural language processing (NLP) tasks, has attracted considerable attention. As a large number of various types of entities widely exist in clinical texts, studies on entity recognition from clinical texts cover clinical entity recognition, clinical event recognition, protected health information recognition (PHI), etc. Compared to entity recognition in the newswire domain, studies on entity recognition in the clinical domain are slower initially.

The early entity recognition systems in the clinical domain are mainly rule-based, such as MedLEE [1], SymText/MPlus [2, 3], MetaMap [4], KnowledgeMap [5], cTAKES [6], and HiTEX [7]. In the past several years, lots of machine learning-based clinical entity recognition systems have been proposed, may due to some publicly available corpora provided by organizers of some shared tasks, such as the Center for Informatics for Integrating Biology & the Beside (i2b2) 2009 [8], 2010 [9–13], 2012 [14–18] and 2014 track1 [19–23] datasets, ShARe/CLEF eHealth Evaluation Lab (SHEL) 2013 dataset [24], and SemEval (Semantic Evaluation) 2014 task 7 [25], 2015 task 6 [26] 2015 task 14 [27], and 2016 task 12 [28] datasets. The main machine learning algorithms used in these systems are those once widely used for entity recognition in the newswire domain, including support vector machine (SVM), hidden markov model (HMM), conditional random field (CRF) and structured support vector machine (SSVM), etc. Among the algorithms, CRF is the most popular one. Most state-of-the-art systems adopt CRF. For example, in the 2014 i2b2 de-identification challenge, 6 out of 10 were based on CRF, including all top 4 systems. The key to the CRF-based systems lies in a variety of features, which are time-consuming.

In recent years, deep learning, which has advantages in feature engineering, has been widely introduced into various fields, such as image processing, speech recognition and NLP, and has shown great potential. In the case of NLP, deep learning has been deployed to tackle machine translation [29], relation extraction [30], entity recognition [31–35], word sense disambiguation [36], syntax parsing [37, 38], emotion classification [39], etc. Most related studies are limited to the newswire domain rather than other domains such as the clinical domain.

In this study, we comprehensively investigate entity recognition from clinical texts based on deep learning. Long-short term memory (LSTM), a representative variant of one type of deep learning method (i.e., recurrent neural network [40]), is deployed to recognize clinical entities and PHI instances in clinical texts. Specifically, we investigate the effects of two different types of character-level word representations on LSTM when they are used as parts of input of LSTM, and compare LSTM with CRF and other state-of-the-art systems. Experiments conducted on corpora of the 2010, 2012 and 2014 i2b2 NLP challenges show that: 1) each type of character-level word representation is beneficial to LSTM on entity extraction from clinical texts, but it is not easy to determine which one is better. 2) LSTM achieves highest micro-average F1-scores of 85.81% on the 2010 i2b2 medical concept extraction, 92.29% on the 2012 i2b2 clinical event detection, and 94.37% on the 2014 i2b2 de-identification, which outperforms CRF by 2.12%, 1.47% and 1.79% respectively. 3) Compared with other state-of-the-art systems, the LSTM-based system is considerably competitive.

The following sections are organized as: section 2 introduces RNN in detail, experiments and results are presented in section 3, section 4 discusses the experimental results and section 5 draws conclusions.

## Methods

**Fig. 1:**
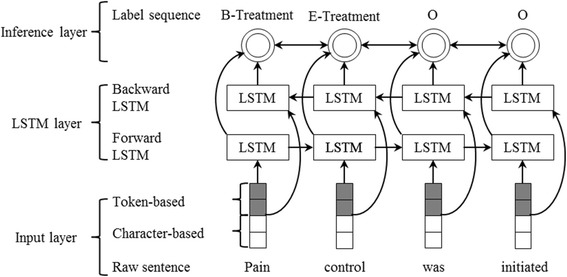
Overview architecture of our LSTM

Entity recognition is usually treated as a sequence labeling problem, which can be modeled by RNN. Instead of traditional RNN, we used Long short-term memory (LSTM) [41, 42], a variant of RNN that is capable of capturing long-distance dependencies of context and avoiding gradient varnishing or exploding [43, 44], for entity recognition from clinical texts. The overview architecture of the LSTM used in our study is shown in Fig. 1, which consists of the following three layers: 1) input layer - generates representation of each word of a sentence using dictionary lookup, which includes two parts: token-level representation (denoted by grey squares) and character-level representation (denoted by blank squares); 2) LSTM layer – takes the word representation sequence of the sentence as input and returns another sequence that represents context information of the input at every position; 3) Inference layer – makes tagging decisions according to the output of the LSTM layer, that is, outputting a label sequence. Before introducing each the three layers one-by-one in detail, we present the LSTM unit first as it is used in both input layer and LSTM layer.

### LSTM unit

**Fig. 2:**
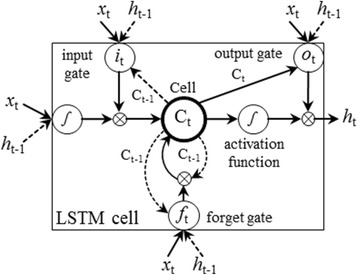
Structure of an LSTM unit

A LSTM unit is composed of three multiplicative gates: an input gate, a forget gate and an output gate, which control the proportion of input information transferred to a memory cell, the proportion of historical information from the previous state to forget, and the proportion of output information to pass on to the next step respectively. Fig. 2 gives the basic structure of an LSTM unit at step *t* that takes *x*_t_, *h*_t-1_ and *c*_t-1_ as input and produces *h*_t_ and *c*_t_ via the following formulas:

$$ \begin{array}{l}{i}_t=\sigma \left({W}_{x i}{x}_t+{W}_{h i}{h}_{t-1}+{W}_{c i}{c}_{t-1}+{b}_i\right)\hfill \\ {}{f}_t=\sigma \left({W}_{x f}{x}_t+{W}_{h f}{h}_{t-1}+{W}_{c f}{c}_{t-1}+{b}_f\right)\hfill \\ {}{c}_t = {f}_t\odot {c}_{t-1}+{i}_t\odot tanh\left({W}_{x c}{x}_t+{W}_{h c}{h}_{t-1}+{b}_c\right)\hfill \\ {}{o}_t=\sigma \left({W}_{x o}{x}_t+{W}_{h o}{h}_{t-1}+{W}_{c o}{c}_t+{b}_o\right)\hfill \\ {}{h}_t = {o}_t\odot tanh\left({c}_t\right),\hfill \end{array} $$

where *σ* is the element-wise sigmoid function, ☉is the element-wise product, *i*_*t*_, *f*_*t*_ and *o*_*t*_ are the input, forget, and output gates, *c*_*t*_ is the cell vector, *W*_*i*_, *W*_*f*_, *W*_*c*_, *W*_*o*_ (with subscripts: *x*, *h* and *c*) are the weight matrices for input *x*_*t*_, hidden state *h*_*t*_ and memory cell *c*_*t*_ respectively, and *b*_*i*_, *b*_*f*_, *b*_*c*_ and *b*_*o*_ denote the bias vectors.

### Input layer

**Fig. 3:**
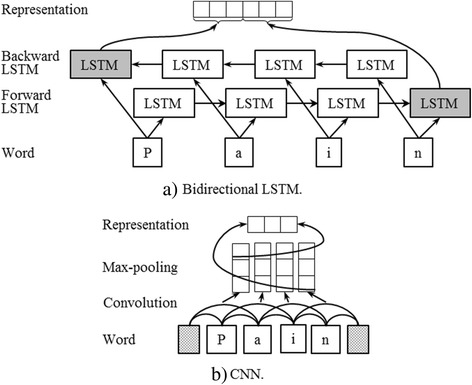
Character-level representation generation models. **a** Bidirectional LSTM. **b** CNN

The representation of a word is generated from the following two aspects: token-level and character-level, which capture context information and morphological information of the word respectively. The token-level representation is usually pre-trained by neural language models, such as continuous bag-of-words (CBOW) and skip-gram [45], on a large unlabeled data. To generate character-level representation, we can use a bidirectional LSTM, which can capture both past and future contexts of words, or a convolutional neural network (CNN) to model the character sequences of words (see Fig. 3). In the bidirectional LSTM (see Fig. 3a), the last two output vectors of the forward and backward LSTMs (rectangles in grey) are concatenated into the character-level representation of the word (i.e., pain). In the CNN (see Fig. 3b, where chess boards are paddings), the sequence of character embeddings are convoluted with filters and further pooled to generate the character-level representation of the word (i.e., pain). For detailed information about CNN, please refer to [46].

### LSTM layer

A bidirectional LSTM is used to generate context representation at every position. Given a sentence *s = w*_1_ *w*_2_ *…w*_*n*_ with each word *w*_*t*_ (1 ≤ *t* ≤ *n*) represented by *x*_*t*_ (i.e., concatenation of token-level and character-level representations of the word), the bidirectional LSTM takes a sequence of word representations *x = x*_1_ *x*_2_ *…x*_*n*_ as input and produces a sequence of context representations *h = h*_1_ *h*_2_ *…h*_*n*_, where *h*_*t*_ = [*h*_*ft*_^T^, *h*_*bt*_^T^]^T^ (1 ≤ *t* ≤ *n*) is a concatenation of outputs of both forward and backward LSTMs.

### Inference layer

Conditional random field (CRF) is employed to predict a label sequence from a sequence of context representations. Given a training set *D =* {(*x* ^(*i*)^, *y* ^(*i*)^)| *i* = 1,…,*m*} (*y* ^(*i*)^ is a label sequence like “… O B-problem I-problem O …” for clinical entity recognition), all parameters of CRF (*θ*) are estimated by maximizing the following log-likelihood function over *D* (only 1^st^ order is considered here):

1 $$ L\left(\theta \right)={\displaystyle {\sum}_{i=1}^m} \log p\left({y}^{(i)}\Big|{x}^{(i)},\theta \right), $$

where

$$ \begin{array}{l} p\left({y}^{(i)}\Big|{x}^{(i)},\theta \right)= p\left({y}^{(i)}\Big|{h}^{(i)},\theta \right)\\ {}=\frac{ \exp \left({\displaystyle {\sum}_{t=1}^n}{\theta}_{y_{t-1}^{(i)}{y}_t^{(i)}}^T{h}_t^{(i)}\right)}{{\displaystyle {\sum}_{y^{\hbox{'}}\in Y\left({x}^{(i)}\right)}} \exp \left({\displaystyle {\sum}_{t=1}^n}{\theta}_{y_{t-1}^{\hbox{'}}{y}_t^{\hbox{'}}}^T{h}_t^{(i)}\right)}\end{array} $$

*Y*(*x* ^(*i*)^) denotes the set of possible label sequences for *x* ^(*i*)^.

The goal of inference at test phase is to search the label sequence y* with the highest conditional probability:

2 $$ {y}^{*}=\underset{y\in Y(x)}{\mathrm{argmax}} p\left( y\Big| x,\theta \right)=\underset{y\in Y(x)}{\mathrm{argmax}} p\left( y\Big| h,\theta \right) $$

Equation 1 and equation 2 can be solved efficiently by dynamic programing and the Viterbi algorithm respectively.

It is clear that if interactions between successive labels are not considered, the inference layer will be simplified into a softmax output layer to classify each token individually.

## Results

In order to investigate the performance of LSTM on entity recognition from clinical texts, we start with two baseline systems: 1) a CRF-based system using rich features (denoted by CRF); 2) a LSTM-based system only using token-level word representations in the input layer (denoted by LSTM-BASELINE), then compare them with the LSTM-based systems using token-level word representations and two different types of character-level word representations. Moreover, we also compare the LSTM-based systems with other state-of-the-art systems. Three benchmark datasets from three clinical NLP challenges: i2b2 (the Center for Informatics for Integrating Biology & the Beside) 2010, 2012 and 2014 are used to evaluate the performance of all systems. Both 2010 and 2012 i2b2 NLP challenges have a subtask of clinical entity recognition, and the 2014 i2b2 NLP challenge have a subtask of PHI recognition.

### Datasets and evaluation

Three types of clinical entities, namely problem, test and treatment, require to be recognized in the 2010 i2b2 NLP challenge, while six types of clinical entities, namely problem, test, treatment, department, evidential and occurrence, in the 2012 i2b2 NLP challenge. In the 2014 i2b2 NLP challenge, seven types of PHI need to be recognized. The detailed statistics of the entity recognition datasets of the three challenges are listed in Table 1, where “2010”, “2012” and “2014” denote the i2b2 NLP challenges in corresponding years, and “#*” denotes the number of ‘*’.

**Table 1:** Statistics of entity recognition datasets used in our study

Challenge		2010	2012	2014
Training	#Note	349	190	790
	#Entity	27837	16468	17405
Test	#Note	477	120	514
	#Entity	45009	13594	11462

The performances of all systems are measured by micro-averaged precision (P), recall (R) and F1-score (F) under different criteria, which are calculated by the official evaluation tools provided by the organizers of the challenges. A brief introduction of the evaluation criteria for the three entity recognition tasks is presented in Table 2, where the key criteria are marked with “*”.

**Table 2:** Evaluation criteria for the three entity recognition tasks

Challenge	Criterion	Remarks
2010	Exact^*^	Entities have the same boundary and same type.
	Inexact	Entities overlap and have the same type.
2012	Span^*^	Entities overlap
	Type	Entities overlap and have the same type.
2014	Exact^*^	Entities have the same boundary and same type.
	Token	“Exact” criterion at token-level.

*represent﻿s the primary evaluation criterion for each task

### Experimental settings

Before training LSTM, we use the following two simple rules to split raw texts into sentences and tokenize the sentences:

1. Sentence split: separate sentences using ‘\n’, ‘.’, ‘?’ and ‘!’.
2. Tokenization: split sentences into tokens by blank characters at first, and then separate those tokens composed of more than two types of characters (letters, digitals and other characters) into smaller parts that only contains only one type of characters. For example, “4/16/91CPT Code:” is split into “4/16/91CPT” and “Code:” at first, and then further separated into ‘4’, ‘/’, “16”, ‘/’, “91”, “CPT”, “Code” and ‘:’.

In this study, we use “BIOES” (B-beginning of an entity, I-insider an entity, O-outsider an entity, E-end of an entity, S-a single-token entity) to represent entities, and follow previous studies [31–35] to use the stochastic gradient descent (SGD) algorithm for parameter estimation with hyperparameters as shown in Table 3. The token-level word representations are pre-trained by word2vec [45] on a large-scale unlabeled dataset from MEDLINE and Wikipedia, and the character representations are randomly initialized from a uniform distribution ranging in [-1, 1]. Both token-level word representations and character representations are fine-tuned during training. We adopt CRFsuite [47] as an implement of CRF, and the features used in the CRF-based system includes bag-of-words, part-of-speech, combinations of words and POS tags, word shapes, affixes, orthographical features, sentence information, section information, general NER information, and dictionary features. All model parameters are optimized by 10-fold cross validation on training datasets.

**Table 3:** Hyperparameters chosen for all our experiments

Hyperparameter	2010/2012/2014
Dimension of token-level word representation	50
Dimension of character representation	25
Character-level LSTM size	25
Character-level CNN filter size	3
Character-level CNN filter number	25
Token-level LSTM size	100
Dropout probability	0.5
Learning rate	0.005
Gradient clipping	5.0
Training epochs	50/30/55

### Experimental results

LSTM only using token-level word representations as input (i.e., LSTM-BASELINE) achieves F1-scores of 85.36% and 92.58% under “exact” and “inexact” criteria on the 2010 i2b2 challenge test set, F1-scores of 92.20% and 87.74% under “span” and “type” criteria on the 2012 i2b2 challenge test set, and F1-scores of 93.30% and 96.05% under “exact” and “token” criteria on the 2014 i2b2 challenge test set, as shown in Table 4, much higher than CRF. The key performance measure differences between LSTM-BASELINE and CRF on the three test sets are 1.67%, 1.38% and 0.72%, respectively.

**Table 4:** Performances of LSTM and CRF-based models for the three tasks (F1-score %)

Model	2010 i2b2 challenge (Concept Extraction)		2012 i2b2 challenge (Event Detection)		2014 i2b2 challenge (De-Identification)	
	Exact	Inexact	Span	Type	Exact	Token
CRF	83.69	91.39	90.82	83.72	92.58	95.37
LSTM-BASELINE	85.36	92.58	92.20	87.74	93.30	96.05
LSTM + char-LSTM	85.81	92.91	92.29	86.94	94.29	96.54
LSTM + char-CNN	85.65	92.77	92.25	87.66	94.37	96.67
LSTM + char-LSTM + CNN	85.78	92.76	92.28	87.80	94.16	96.44

When one type of character-level word representations (i.e., character-level word representations generated by LSTM or CNN, denoted by char-LSTM and char-CNN respectively in Table 4) is added in the input layer as shown in Fig. 1, the performance of LSTM is slightly improved, LSTM considering char-LSTM (i.e., LSTM + char-LSTM) achieves a little better performance on the 2010 and 2012 i2b2 NLP challenge test sets, while the LSTM considering char-CNN (i.e., LSTM + char-CNN) achieves a little better performance on the 2014 i2b2 NLP challenge. No remarkable sign shows which character-level word representation is better. When both two types of character-level word representations are added, the performance of LSTM is not further improved. The highest F1-scores of LSTM are 85.81% and 92.91% under “exact” and “inexact” criteria on the 2010 i2b2 challenge test set, 92.29% and 86.94% under “span” and “type” criteria on the 2012 i2b2 challenge test set, and 94.37% and 96.67% under “exact” and “token” criteria on the 2014 i2b2 challenge test set.

Moreover, we also compare “LSTM + char-LSTM” with other state-of-art systems including the best systems of the three challenges and the best up-to-date systems on the same corpora (as shown in Table 5, where the starred systems are the best systems of the corresponding challenges). “LSTM + char-LSTM” significantly outperforms the best systems of the three challenges. On the 2010 i2b2 NLP challenge corpus, “LSTM + char-LSTM” achieves almost the same F1-score as the current best system (85.81% vs 85.82%), which is a SSVM-based system using rich hand-crafted features, under “exact” criterion. On other two i2b2 NLP challenge corpora, “LSTM + char-LSTM” outperforms the current best systems.

**Table 5:** Comparison of the performances of various systems on the three tasks (%)

	System	Method	Exact F1-score	Inexact F1-score
2010	LSTM + char-LSTM	RNN	85.81	92.91
	Tang et al (2013) [10]	SSVM	85.82	92.40
	Bruijin et al (2011)^*^ [13]	Semi-Markov	85.23	92.44
	Kim et al (2015) [9]	CRFs	84.30	-
	Jiang et al (2011) [12]	CRFs	83.91	91.30
	System	Method	Span F1-score	Type Accuracy
2012	LSTM + char-LSTM	RNN	92.29	86.94
	Xu et al. (2013)^*^ [15]	CRFs	91.66	85.74
	Tang et al. (2013) [16]	CRFs + SVM	90.13	83.60
	Sohn et al. (2013) [17]	CRFs	87.00	76.77
	Aleksandar et al. (2013) [18]	CRFs	87.29	82.00
	System	Method	Exact F1-score	Token F1-score
2014	LSTM + char-LSTM	RNN	94.29	96.54
	Yang et al. (2015) [20]	CRFs	93.60	96.11
	He et al. (2015) [22]	CRFs	92.32	95.14
	Liu et al. (2015) [21]	CRFs + rule	91.24	94.64
	Dehghan et al. (2015) [23]	CRFs + rule	91.13	95.31

## Discussion

In this study, we investigate the performance of LSTM on entity recognition from clinical texts. The LSTM-based systems achieves highest F1-scores of 85.81% under “exact” criterion on the 2010 i2b2 challenge test set, 92.29% under “span” criterion on the 2012 i2b2 challenge test set, and 94.37% under “exact” criterion on the 2014 i2b2 challenge test set, which are competitive with other state-of-the-art systems. The major advantage of the LSTM-based system is that it does not rely on a large number of hand-crafted features any more. Similar to previous studies in the newswire domain, LSTM shows great potential on entity recognition in the clinical domain, outperforming most traditional state-of-the-art methods that suffer from fussy feature engineering such as CRF.

Experiments shown in Table 4 demonstrate that any one type of the two character-level word representations is beneficial to entity recognition from clinical texts. The reason may lie in that both the two types of character-level word representations have ability to capture some morphological information of each word such as suffixes and prefixes, which cannot be captured by the token-level word representation that relies on word context. Then, when any one of the character-level word representations is added into the input layer of LSTM, errors like “Test” event “URINE” missed in “2014-11-29 05:11 PM URINE” and hospital “FPC” correctly identified in “… have a PCP at FPC …” but missed in “… Dr. Harry Tolliver, FPC cardiology unit …” are fixed.

Although the LSTM-based system shows better overall performance than almost all state-of-the-art systems mentioned in this study, but it does not show better performance on all types of entities. For example, the best system on the 2012 i2b2 challenge corpus (i.e., Xu et al. (2013) [15]) achieves better “span” F1-score than the LSTM-based system on “Test” events (94.16% vs 93.69%). The best system on the 2014 i2b2 challenge corpus (i.e., Yang et al. (2015) [20]) achieves better “exact” F1-score than LSTM-based system on “ID” instances (92.71% vs 91.94%). There are two main reasons: 1) the current LSTM-based system does not use knowledge bases widely existing in the clinical domain, but the other state-of-the-art systems take full advantages of them; 2) although the character-level word representation has ability to capture some morphological information of each word, it cannot cover morphological information of specific words such as fixed size digitals. Therefore, there are two possible directions for further improvement in our opinion: 1) How to integrate widely existing knowledge bases into the input of LSTM; 2) How to use LSTM to recognize entities in specific formats. We will try them in the future.

In recent months, a few studies on deep learning for entity recognition from clinical text are also proposed. For example, Abhyuday et al. proposed two RNN-based models for medical event detection on their own annotated dataset, one of which recognizes medical event detection as a classification problem and the other one as a sequence labeling problem [48, 49]. Both the two RNN-based models adopt traditional RNN, which is not as good as LSTM, and only take token-level word representation as their input. Franck et al. deployed a similar RNN model for the de-identification task on the 2014 i2b2 NLP challenge corpus and the MIMIC dataset [50]. According to the experimental results reported in this study and the similar studies, we may conclude that our LSTM outperforms theirs. For example, the F1-score of the RNN model proposed by Franck et al. on the 2014 i2b2 dataset, as reported, is 97.85% under the binary HIPAA token criterion (only evaluating the HIPAA-defined PHI instances under “token” criterion). Under the same evaluation criterion, the corresponding F1-score of “LSTM + char-LSTM” is 98.05% on i2b2-2014 dataset. The results demonstrate that our LSTM outperforms RNN proposed by Franck et al [50]. Therefore, the results reported in this study can be a new benchmark system based on deep learning methods.

## Conclusions

In this study, we comprehensively investigate the performance of recurrent neural network (i.e., LSTM) on clinical entity recognition and protected health information (PHI) recognition. Experiments on the 2010, 2012 and 2014 i2b2 NLP challenge corpora prove that 1) LSTM outperforms CRF; 2) By introducing two types of character-level word representations into the input layer of LSTM, LSTM is further improved; 3) the final LSTM-based system is competitive with other state-of-the-art systems. Furthermore, we also point out two possible directions for further improvement.
